# Happy Marriages

**Published:** 1860-12

**Authors:** 


### HAPPY MARRIAGES.

All sorts of "old saws" are grunted out as to love and marriage, and how to be happy in domestic life; but very few enter that beatific state, whose first steps were taken in deliberate calculation; still, it would be far better for all concerned, if these first steps had been preceded by a wise deliberation and foresight. In almost all cases, the first "bias" is determined by some physical quality, the face, the foot, the ankle; the twinkle of the eye, the dimple of a cheek, the lisp of the tongue, the port of the head; the length, the richness, the color of a curl, or the general carriage or contour of the body. Mental and high moral qualities command respect; and as to that middle ground between respect and love, that is, admiration, it is excited by the qualities of the heart, such as frankness, nobility of nature, and implicit trustfulness. But for the kindling up of real, old-fashioned, flaming, world-defying, heart-breaking love, the physical properties, in too many cases, have the initiating and predominant agency.

Ill-assorted marriages are in a great number of instances the result of parental remissness, in not beginning early enough to instill into the mind of the child such an aversion to certain traits of character, and such a high estimate of certain moral qualities, as a true wisdom would dictate in the premises.

It certainly is not an impossible thing to impress the youthful mind with an unconquerable repugnance against a character the most striking trait of which is a contemptible trickery, an abhorrent profanity, a little-souled meanness, or a degrading animalism. Just as well may the young heart be fortified against loving the miser, the spenthrift and the gamester—against those whose prominent exhibitions demonstrate an irascibility, an all-absorbing selfishness or stony-heartlessness; or a contempt of honest labor, of religion, or of pecuniary obligation. While our children may be early taught an aversion to such traits of character, their admiration may be cultivated for all that is manly and honorable and self-sacrificing; for all that is true and pure and generous; for all who are industrious, diligent, and economical.

It is unwise to hope for domestic happiness in the possession of a single favorable trait of character; it is better to look for a combination, and they are to be most congratulated who can discern and woo and win the possessor of the largest number of good points. First of all, the man whom you love, the woman whom you adore, should possess a high sense of right and wrong; next, bodily health; and, thirdly, moral bravery, a courage to be industrious, economical and self-denying. With these three traits, principle, health, and a soul that can do and dare all that one ought to, domestic felicity will abide. None ought to marry who can not command the means of enabling them to live in comfort according to their station in life, without grinding economies.

It is useless to talk about love in a cottage. The little rascal always runs away when there is no bread and butter on the table. There is more love in a full flour-barrel than in all the roses and posies and woodbines that ever grew.

No mechanic should marry until he is master of his trade; nor a professional man, until his income is adequate to the style of life which he determines upon; nor the merchant, until his clear annual gains are equal to his domestic expenditures, unless indeed there are, in either case, independent and unconditional sources of income.

No man ought to marry who has to work like a horse from morning until night to supply family necessaries, whether it be by brain or body; for if the body is thus made a drudge of, it perpetuates impaired power to the race; while if the brain is overwrought, its effects will be seen in children of feeble intellect, if indeed they be not demented. To calculate, therefore, on a reasonable share of domestic enjoyment, the parties most interested should aim to find in each other as great an amount as may be of high moral principle, of bodily health, and either the actual possession of a suitable maintenance, or an individual ability to secure it without peradventure.

